# Epidemiology, Prevention and Clinical Treatment of Allergic Rhinitis: More Understanding, Better Patient Care

**DOI:** 10.3390/jcm11206062

**Published:** 2022-10-14

**Authors:** Yang Liu, Zheng Liu

**Affiliations:** 1 Department of Otolaryngology-Head and Neck Surgery, Tongji Hospital, Tongji Medical College, Huazhong University of Science and Technology, Wuhan 430030, China; 2 Institute of Allergy and Clinical Immunology, Tongji Hospital, Tongji Medical College, Huazhong University of Science and Technology, Wuhan 430030, China; 3 Hubei Clinical Research Center for Nasal Inflammatory Diseases, Wuhan 430030, China

Allergic rhinitis (AR) is a noninfectious inflammatory disease of the nasal mucosa mediated by IgE after atopic individuals are exposed to inhaled allergens and involving a variety of immune cells and cytokines [1]. AR is characterized by nasal obstruction, rhinorrhea, sneezing and nasal pruritus [1]. AR exacerbates asthma, and most asthmatics suffer from AR [1]. AR is highly prevalent worldwide, and the clinical management of AR is yet to be improved. Thus, AR imposes significant burden on individuals and society. This editorial will focus on recent advances on the epidemiology, prevention, and clinical management of AR, as well as future research directions.

With regards to the epidemiology of AR, factors such as prevalence, disease classification, allergen sensitization, co-morbidities, risk factors, genetic susceptibility, and costs, etc., are widely discussed [2]. Globally, the prevalence of AR remains increasing, particularly in low- and middle-income countries, and ranges from 1.0% to 54.5% [2,3]. The prevalence of AR is roughly 5% in children by 3 years of age, increases with age from 8.5% of 6–7 year-olds to 14.6% of 13-14 year-olds, and reaches more than 11.8% to 46% in people aged 20–44 [2]. Notably, the incidence of AR is higher in males than in females before puberty, but this trend reverses after puberty [4]. A systematic review and meta-analysis showed that in children less than 11 years of age, significantly more boys than girls exhibited rhinitis symptoms (male–female ratio 1.21, 95% CI 1.17–1.25), whereas in adolescents (11 to 18 years of age), males were significantly less affected than females (male–female ratio 0.90, 95% CI 0.85–0.95) [4]. A number of risk factors for AR have been identified including antibiotic use, air pollution, and maternal and paternal smoking, etc. [2]. A recent large-scale meta-GWAS on AR of European ancestry identified a total of 41 risk gene loci for AR, including *TLR1*, *TLR10*, *GATA3*, *IL33*, *NFKB1*, etc., which are involved in both innate and adaptive immune pathways and bridge the environmental insults and cell responses [5]. Epidemiological studies on the risk and protective factors of AR in the context of genetic susceptibility and epigenetic modification have produced a lot of evidence [6]. Two major observations regarding the cause of AR are the ‘sibling effect’ and the ‘farm effect’, which lead to the ‘hygiene hypothesis’ and, later, the ‘biodiversity hypothesis’ [6]. Future epidemiological studies are needed to evaluate and refine these hypotheses given the paradigm shift from atopic sensitization to barrier dysfunction. Encouragingly, there are more options than ever before for the characterization of environmental and other factors, such as “omics” technology in microbiology and metabolism [6].

With respect to prevention, graded prevention of AR has been proposed [7]. Primary prevention involves eliminating causes that are critical to disease development, including changing causal or predisposing factors related to the environment and the workplace [7]. Secondary prevention includes the avoidance of clinically relevant allergens and irritants. Tertiary prevention aims to prevent exacerbations, thereby improving disease control and reducing medication needs, where appropriate, pharmacological prophylaxis and allergen-specific immunotherapy for patients with early symptoms [7,8]. In the future, carefully designed and executed randomized trials are needed to properly measure and report the effects of the above measures on AR prevention and potential adverse effects, so as to reduce the overall risk of bias. In addition, a correct assessment of any long-term adverse effect will require large observational studies.

Allergen avoidance, drug therapy and allergy immunotherapy (AIT) are the main clinical intervention modalities for AR [2]. The first-line treatments for AR include H1 antihistamines, intranasal corticosteroids (INCS) and a combination of INCS and H1 antihistamines, depending on the severity of the patient’s symptoms [2]. H1 antihistamines may be used in patients with mild symptoms or who do not want to use INCS [2]. For those with persistent or moderate to severe symptoms, INCS is recommended [2]. Fixed combinations of INCS and H1 antihistamines are mainly used in patients who do not benefit from INCS alone [2]. The stepwise treatment is suggested for AR. Zhu RF et al. found that the Allergic Rhinitis Control Test Questionnaire is valuable in guiding the step-up and step-down pharmacotherapy of AR, which can significantly improve disease control and reduce the medication use [9]. Recently, Chinese medicine has attracted attention in treating AR, since it has been shown to relieve symptoms [10,11]. Pharmacotherapy shows effectiveness in most patients; however, many patients have poor medication adherence [2]. Mobile-phone-based apps and short message services are effective methods to improve adherence to medical therapy in AR patients [12]. Recent evidence has shown that acupuncture was effective in alleviating symptoms in AR patients [13]; however, the mechanism of action remains undefined. 

Available asthma biologics targeting type 2 response, such as omalizumab, an anti-IgE antibody and dupilumab targeting the IL-4/IL-13 pathway, have been attempted in patients with AR. Two meta-analyses revealed that omalizumab was effective and safe to treat uncontrolled AR patients [14,15]. Although type 2 biologics have produced promising efficacy for the treatment of AR [15,16], they are more reasonably reserved for patients with uncontrolled disease given their high cost [16,17,18]. Biologics have also been used in patients under AIT to reduce adverse events and improve efficiency of AIT. Corren J et al. found that the simultaneous use of dupilumab reduced the epinephrine rescue treatment rate from 19.2% to 7.7% in grass-pollen-induced AR patients under subcutaneous AIT [19]. 

AIT is considered as the only etiological and most effective therapy for AR [20]. However, AIT has a number of limitations, including high cost, systemic side effects, the long treatment period and the absence of biomarkers to monitor and predict therapeutic efficacy [21]. Recently, instead of conventional T helper and regulatory cells, type 2 follicular helper T (TFH2) cells and follicular regulatory T (TFR) cells have been identified as the real cell types regulating B cell IgE production in AR [22,23]. AIT has been found to increase the number and function of TFR cells and re-establish the balance between TFH2 and TFR cells in AR [22,23]. The changes in TFH2 and TFR cells after AIT associate with AIT efficacy [22,23]. These studies suggest that TFH and TFR cells may be targeted to improve AIT efficacy and serve as biomarkers for AIT efficacy. In the future, more investigations are required to validate the use of TFH and TFR cells as biomarkers for AIT treatment, and their potential value as therapeutic targets for AR. It is hoped that advances in immunotherapy technology will achieve greater efficacy and safety. AIT administration routes, adjunctive therapies, vaccine adjuvants and novel vaccine technologies are being pursued to achieve this goal [24,25,26]. Intra-lymphatic immunotherapy, component-resolved AIT, recombinant proteins, nanoparticles and virus-like particle vaccines, T- and B-cell peptides and allergoids are potential options to improve AIT effectiveness and reduce side effects.

A major outstanding question is, when the above therapies fail, what can we do next? For those patients, surgical operations may be considered. Surgical intervention may aim at reducing nasal obstruction as a means of improving quality of life of AR patients [27]. Other surgical options, such as posterior nasal nerve resection or ablation and vidian neurectomy can reduce the neural reflex and neuro-immune crosstalk in AR patients and therefore relieve symptoms, but unfortunately, some complications can occur [28,29,30]. The long-term effect of surgical interventions and their related side effects should be studied in high-quality clinical trials. 

There are several items that should be addressed in future research efforts for AR. First, to integrate genomics, transcriptomics, proteomics and metabolomics into AR pathogenesis investigation will promote the understanding of the interactions between environmental factors, allergen sensitization and disease development. Second, biomarkers are urgently needed to predict treatment response and tailor the management of AR patients. Third, research into novel vaccines and improvement of treatment schemes in AIT is needed to increase safety while maintaining or even enhancing the efficiency of AIT. Vaccines targeting B or T cells have been tried, and some of them have demonstrated promising results [31,32]. Fourth, in the real world, there is still considerable uncertainty about the true effectiveness of AIT after long-time follow-up. To generate real-world evidence for AIT to treat AR will provide necessary complementary evidence to existing randomized controlled trials. Fifth, to develop monoclonal antibodies to allergens may hold promise for AR patients who respond moderately or poorly to AIT. In a recent phase1b study, Shamji MH et al. reported that a single, passive-dose administration of Fel d 1-neutralizing IgG antibodies improved nasal symptoms in cat-allergic patients and suppressed nasal type 2 allergic response [33]. 

The present Special Issue invites authors to contribute original research articles as well as review articles related to all aspects of AR in epidemiology, prevention and clinical treatment. We hope that our readers will enjoy this issue of the *Journal of Clinical Medicine*, which extends our knowledge of AR and gives us the confidence to fight against this highly prevalent disorder. 
